# Capturing the multifaceted function of adipose tissue macrophages

**DOI:** 10.3389/fimmu.2023.1148188

**Published:** 2023-02-16

**Authors:** Alyssa J. Matz, Lili Qu, Keaton Karlinsey, Anthony T. Vella, Beiyan Zhou

**Affiliations:** ^1^ Department of Immunology, School of Medicine, University of Connecticut, Farmington, CT, United States; ^2^ Institute for Systems Genomics, University of Connecticut, Farmington, CT, United States

**Keywords:** obesity, adipose tissue, macrophage, immune response, single-cell RNA sequencing

## Abstract

Adipose tissue macrophages (ATMs) bolster obesity-induced metabolic dysfunction and represent a targetable population to lessen obesity-associated health risks. However, ATMs also facilitate adipose tissue function through multiple actions, including adipocyte clearance, lipid scavenging and metabolism, extracellular remodeling, and supporting angiogenesis and adipogenesis. Thus, high-resolution methods are needed to capture macrophages’ dynamic and multifaceted functions in adipose tissue. Herein, we review current knowledge on regulatory networks critical to macrophage plasticity and their multifaceted response in the complex adipose tissue microenvironment.

## 1 Introduction

Obesity is a prevalent health risk to an expanding list of co-morbidities, increasing global rates of disability and mortality (1–3). Obesity induces white adipose tissue (WAT) dysfunction that significantly contributes to obesity-associated health risks through chronic, low-grade tissue inflammation, insulin resistance, hyperlipidemia, and hypertension (4). Studies have demonstrated that controlling obesity-associated WAT inflammation can improve tissue function and systemic health (5–9).

Within white adipose tissue, macrophages are the most abundant immune population. During obesity, the adipose tissue macrophage (ATM) population increases 10-fold in cell number and exacerbates local inflammation. ATMs originate from hematopoietic stem-cell-derived circulating monocytes and self-replicating tissue residents seeded during fetal development (10–12). Inhibiting macrophage expansion by limiting monocyte-derived macrophage recruitment during obesity lessens WAT inflammation (11, 13, 14). However, the ATM population is heterogeneous and critical to tissue function. Indeed, inhibition of specific ATM functions worsens systemic metabolic health (15). Thus, it is essential to understand the molecular signaling pathways that enrich beneficial ATM functions under obesity rather than eliminate them. Several ATM functions have been identified, including dead adipocyte clearance, lipid scavenging and metabolism, extracellular remodeling, and supporting angiogenesis and adipogenesis. In addition, the macrophage population in brown adipose tissue is less defined, but several studies demonstrate their importance in maintaining thermogenesis (16–18). This review summarizes ATM functions important to white adipose tissue and relevant regulatory pathways that promote these actions. Herein, we aim to demonstrate the power of high-resolution investigations to characterize diverse macrophage populations and the need for function-based analysis to deconvolute targetable networks to lessen obesity-induced comorbidity and mortality.

## 2 Macrophage diversified responses

**Figure 1:**
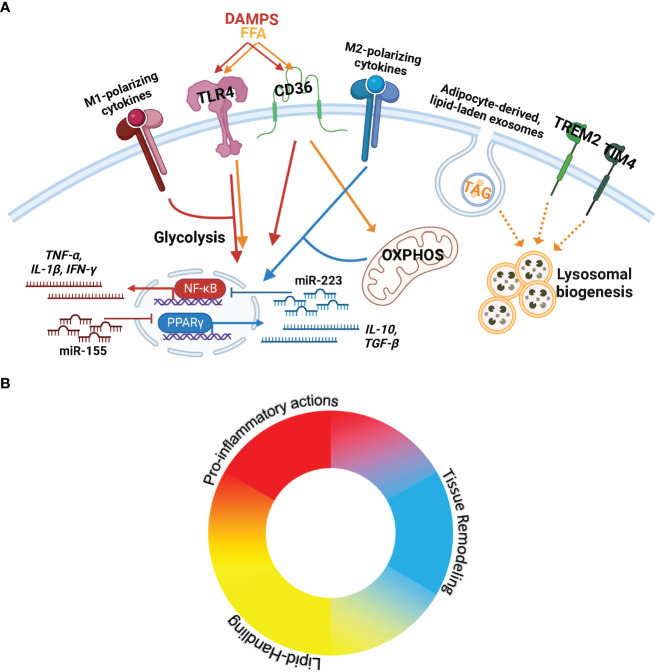
ATMs experience a complex microenvironment with opposing and compounding signals that results in a spectrum of activity. **(A)** Key stimuli, receptors, and signaling cascades known to control macrophage actions and implicated in ATM biology. (From Left to Right) M1-polarizing cytokines include TNF-α and IFN-γ. M1-polarizing chemokines are also present in AT and include CCL2, CCL4, and CXCL8. DAMPS= Danger-associated molecular patterns including exogenous (lipopolysaccharide) and endogenous signaling (e.g. apoptotic cells) are recognized through TLR4 to elicit pro-inflammatory signaling (JAK-STAT/NK-κB/MAPK) towards the production of type-1 cytokines (TNF-α, IL-1β, IFN-γ). miR-155 promotes M1-polarization through inhibition of signaling cascades towards PPARγ. FFA= Free Fatty Acids signaling through TLR4 elicits pro-inflammatory activation; CD36 transports FFAs, upregulating fatty acid oxidation and OXPHOS= Oxidative Phosphorylation. CD36 also recognizes DAMPs to activate pro-inflammatory activation. M2-polarizing cytokines (IL-4, IL-13, IL-10, IL-17A, IL-25) activate PPARγ to produce type-2 cytokines (IL-10, TGF-β). miR-223 promotes M2-polarization through the repression of pro-inflammatory signal cascade components. OXPHOS is required for M2 actions. Adipocyte-derived exosomes transport neutral lipids (including TAG=Triacylglycerols) into macrophages, which induce lysosomal biogenesis for lipid metabolism. Trem2 and Tim4 also elicit lysosomal biogenesis, but the ligands and downstream signaling cascades are unclear. **(B)** Macrophages are plastic, responding to diverse stimuli to provide appropriate action. Due to the plasticity of macrophages, their multifaceted capacity, and the complex microenvironment in AT, a single ATM can perform various actions. The ATM population is best represented as a spectrum of macrophage functions.

Macrophages can perform an array of functions to diverse stimuli, including pathogen- and damage-associated molecular patterns, cytokines, chemokines, metabolites, and extracellular vesicles (19, 20). Several models have been developed to classify macrophage features. One of the most widely utilized models is the M1/M2 paradigm, which delineates two central functional states: classically activated macrophages (M1) and alternatively activated macrophages (M2) (21). This and other *in vitro* models have allowed the characterization of regulatory mechanisms and signaling pathways crucial to several macrophage responses. It is now appreciated that these *in vitro*-based models do not recapitulate the complex stimuli experienced by tissue-resident macrophages but provide a basis to deconvolute the responses seen *in vivo.* Thus, mapped macrophage responses to stimuli experienced in adipose tissue and other physiologic and pathogenic states will be summarized in the following section (**Figure 1A**).

### 2.1 Cytokines and chemokines regulating macrophage actions

WAT is a source of adipokines, including immune modulatory cytokines, chemokines, and growth hormones. Many *in vitro* studies have detailed cytokines/chemokines that differentially contribute to the polarization of macrophages. Tumor necrosis factor alpha (TNF-α), interferon gamma (IFN-γ), C-C motif chemokine 2 (CCL2), CCL4, and interleukin 8 (CXCL8) can induce the classical activation of macrophages. M1-polarized macrophages provide acute pro-inflammatory effector functions by expressing reactive oxygen species, nitric oxide, and secretion of type-1 cytokines such as TNF-α, IFN-γ, and interleukin 1 beta (IL-1β). In contrast, macrophages are activated towards M2 polarization by IL-4, IL-13, IL-10, IL-17A, IL-25, and CCL5 to resolve acute inflammation and secrete type-2 cytokines, including IL-10 and arginase (21–25). The effect of cytokines on the polarization of macrophages has been investigated through *in vivo* studies in various contexts relevant to ATM functions (22, 26–28). Administration of IL-25 to obese mice mitigated weight gain through enhanced ATM M2-polarization, mitochondrial respiratory capacity, and lipolysis, demonstrating the therapeutic potential of targeting macrophage actions in obesity-associated disease (22).

### 2.2 Pattern recognition receptors relevant to ATMs

Macrophages are armed with an extensive repertoire of germ-line encoded pattern recognition receptors (PRRs) that recognize conserved molecular patterns that stimulate rapid innate immunity. Macrophages are also phagocytic and respond to particulate ligands from engulfed vesicles. Macrophage PRRs and ligands are summarized elsewhere (29); herein, we focus on those implicated in adipose tissue function. Receptor ligand interactions regulate macrophage responses *via* the release of stored mediators, transcription activation, and metabolic reprogramming. An important family of PRRs is Toll-like receptors (TLRs), which elicit pro-inflammatory responses to exogenous and endogenous molecular targets. TLR4 can be activated by lipopolysaccharide to initiate classical macrophage polarization (M1), as well as saturated fatty acids (30, 31), which are rich in obese AT. Inhibiting TLR4 signaling in models of obesity improves systemic metabolic function (32). Macrophages also express phosphatidylserine recognition receptors, scavenger receptors, type 3 complement receptors (CR3), β-glucan receptors, Fc receptors, and mannose receptors. Phosphatidylserine recognition receptors are a diverse group of proteins apt for recognizing apoptotic cells (33). Their dynamics in ATMs warrant further investigation, given their role in adipocyte clearance. Within scavenger receptors, CD36 is elevated in ATMs under obese stress (34). CD36 is a receptor for long-chain fatty acid transport (35) and binds various danger-associated molecular patterns (DAMPS) (31, 36, 37). Ligand-dependent activation of signaling cascades through CD36 relies on specific co-receptors and partners (33). Fatty acid binding to CD36 upregulates fatty acid oxidation (38), while DAMP interactions initiate a pro-inflammatory cascade in macrophages (39). CD36-mediated triacylglycerol uptake is necessary to support M2 activation through stimulating lipolysis and elevated oxidative phosphorylation (40). In the case of circulating macrophages, CD36 binding to oxidized low-density lipoproteins initiates a cellular metabolic shift to activate Mitogen-activated protein kinases (MAPK) signaling towards M1-polarization (39, 41, 42).

### 2.3 Key signaling pathways in macrophage function

Various stimuli elicit common downstream signaling pathways and metabolic programs, yielding similar macrophage responses. These signaling cascades’ strength is tuned by small non-coding RNA molecules termed microRNA (miRNAs). The effect of miRNA on the polarization of

macrophages and its impact on ATM function has been extensively researched and is reviewed elsewhere (43). Notably, miR-155 and miR-223 are key regulators of macrophage polarization and profoundly impact systemic metabolism in obesity.

M1 polarization relies on the Janus kinase-signal transducer and activator of transcription (JAK-STAT) pathway *via* nuclear factor kappa B (NF-κB and MAPK signaling (44). The microRNA miR-155 supports pro-inflammatory activation of macrophages (45, 46) by repressing the translation of potent anti-inflammatory mediators including *Socs1, Ship1*, and *IL13Rα1* (47–50).

In contrast, M2 activation depends on the transcription factor Peroxisome proliferator-activated receptor γ (PPARγ), activated downstream of IL4-IL4R signaling. PPARγ stimulates miR-223 expression, supporting PPARγ in a positive feedback loop (51–53). miR-223 suppresses components of NF-κB signaling, including *Nfat5, Rasa1*, and *PKNOX1*, therein reducing pro-inflammatory cytokine production (51–53). Obese mice with systemic deletion of miR-223 had heightened WAT pro-inflammatory macrophage infiltration, inflammation, and worsened systemic metabolism (51).

### 2.4 Macrophage cellular metabolism

Macrophage cellular metabolism is integral to activation and function. For pro-inflammatory activation, macrophages rely on HIF1α-mediated aerobic glycolysis enrichment (40, 54, 55), increasing glucose and oxygen consumption likely to increase the production of reactive oxygen species (40). M2-polarization requires lipolysis and oxidative phosphorylation (56); inhibiting metabolic reprogramming severely weakens M2-mediated responses, including clearance of parasite helminth infections (56). Macrophage lipid metabolism is regulated through lipid uptake, synthesis, and clearance through mediators Sterol regulatory element-binding protein 1 (SREBP) and L-xylulose reductase (LXR). Inhibition of SREBP signaling in ATMs reduces cholesterol efflux, activating M1-polarization cascades and increasing the proportion of pro-inflammatory ATMs (57). In addition, LXR-stimulated fatty acid synthesis represses pro-inflammatory cytokine production in ATMs and increases systemic insulin sensitivity in obesity (58). However, TLR4-mediated signaling inhibits LXR-mediated fatty acid synthesis and downstream Myeloid differentiation primary response protein (MyD88)- and TIR domain-containing adapter molecule (TRIF)- signaling pathways alters the lipid composition of macrophages to intensify inflammation (59). These opposing signaling cascades are likely responsible for the spectrum of ATMs activation states observed *in vivo* (**Figure 1B**).

## 3 Macrophage actions in white adipose tissue

ATM populations are heterogeneous, containing specialized subsets that can perform pro-inflammatory actions, lipid scavenging and metabolism, extracellular remodeling, and support angiogenesis and adipogenesis (**Table 1**). Functional characterization of tissue-resident macrophages is necessary to understand their regulation and identify modulatory pathways to promote metabolic health in obesity.

**Table 1:** Macrophage functions in adipose tissue and identified markers/features of specialized cells.

Adipocyte Tissue Macrophage Functions	Key features	Citation
Pro-inflammatory functions (Dead adipocyte clearance, Immune activation)	CD9+, TNF-α expression	(15, 60, 61)
Lipid-Handling	Tim4+, Abca1+, Lyve-1+ (Lean) Trem2+, CD9+ (Obese)	(34, 62)
Extracellular Remodeling and Angiogenesis	Lyve-1+, MMP-9,-12 expression	(63)
Adipogenesis	Osteopontin expression	(61, 64)

While delineated features and markers have been identified for cells that are best equipped to perform these functions, macrophages are multifaceted and plastic in nature and likely perform multiple actions.

### 3.1 Pro-inflammatory actions

A primary function of recruited macrophages in WAT is immune activation for the removal of dead adipocytes, accomplished by pro-inflammatory ATMs. ATMs produce pro-inflammatory mediators that bolster tissue and systemic inflammation including TNF-α, IL-6, IL-1β, CCL2, Inducible nitric oxide synthase (iNOS), and others (65). Under obesity stress, WAT remodeling is continual, and the macrophage per adipocyte ratio increases significantly (10, 60, 66). The first characterization of ATM dynamics in lean and obese conditions found a shift from M2-like to M1-like cell predominance under obesity and secrete elevated TNF-α (60, 61). RNA-sequencing identified CD9-expressing, pro-inflammatory ATMs that surround dead adipocytes are abundant in obesity (15). ATMs also contribute to local tissue immune activation through antigen presentation to resident adaptive immune cells. During obesity, ATM express increased major histocompatibility complex II (MHCII) and co-stimulatory molecules to activate CD4+ T cells (67).

Notably, glucagon-like peptide-1 (GLP-1) agonists and GLP-1 analogs are a class of medication utilized in the treatment of obesity and type 2 diabetes (68, 69). Administration significantly reduces fat mass and macrophage per gram of fat in models of obesity (70). ATMs and peritoneal macrophages from treated mice also express less TNF-α and IL-6 (70), suggesting a possible direct role on macrophage activity. Given the impact of GLP-1 on obesity-induced health risk, further investigation into their impact on ATM actions is warranted.

### 3.2 Lipid-buffering

Macrophages support adipose tissue storage capacity by metabolizing lipids through lysosomal lipolysis. While all ATMs upregulate surface expression of the CD36 after a high-fat meal (34), subsets of specialized lipid-handling ATMs have been identified in both lean and obese WAT. In lean WAT, a subset of ATMs delineated by phospholipid-transporting ATPase ABCA1 (Abca1), T-cell immunoglobulin and mucin domain-containing protein 4 (Tim4), and lymphatic vessel endothelial hyaluronic acid receptor 1 (Lyve1) expression are self-replicating and most apt for lipid uptake and metabolism (34). After a meal, Tim4+ ATMs increase their lipid uptake, lysosomal content, and release HDL as part of the reverse cholesterol pathway (34).

During obesity, ATM transcriptomes demonstrate lysosome biogenesis is significantly enriched (66). Lipid-laden ATMs are more abundant and associated with elevated lysosome content (15, 62, 66). While Tim4+ ATMs persist in obesity, a novel lipid-laden ATM subset predominates. High-resolution investigations first delineated obesity-associated lipid-laden ATMs with CD9 expression (15), which were further demarcated by Triggering receptor expressed on myeloid cells 2 (Trem2) expression in both mice and humans (62). Recruited monocyte-derived macrophages are programmed by Trem2 signaling into lipid-laden ATMs (15). While inhibiting infiltration of monocyte-derived macrophages into obese WAT lessened overall tissue inflammation (11, 13, 14), specific inhibition of the Trem2 lipid-laden program of recruited macrophages exacerbates dyslipidemia and adipocyte hypertrophy, worsening overall metabolic health (15). Interestingly, ATMs uptake neutral lipids from adipocyte-derived exosomes, a distinct means of lipid handling for macrophages (71). Adipocyte-derived exosomes can induce ATM features from monocytes *in vitro* and, during obesity, are released at a significantly higher rate (71). Tim4 and Trem2 are relevant for ATM lysosome biogenesis. However, their ligand and downstream signaling cascades have not been elucidated. Tim4 and Trem2 are receptors for phosphatidylserine, a major component of exosomes (72, 73). Further research into the mechanism of adipocyte exosomes in developing lipid-laden ATMs could yield translatable discoveries for metabolic disease.

### 3.3 Extracellular remodeling and supporting angiogenesis and adipogenesis

WAT angiogenesis, adipogenesis, and extracellular remodeling are tightly linked processes to expand lipid storage capacity. ATMs regulate each process, but it is unclear if specialized subsets persist in WAT to perform these actions or if multifaceted macrophages contribute to these functions. While these cells do not recapitulate the M2 program, tissue remodeling and Transforming growth factor β (TGF-β) production have been described as an action of M2-polarized macrophages (74, 75). Whether important regulatory pathways towards M2 activation are responsible for ATM extracellular remodeling, angiogenesis, and adipogenesis remains unclear.

Angiogenesis is crucial to prevent hypoxia in expanding WAT. Increasing AT capillary density abrogates obesity-induced insulin resistance and metabolic dysfunction (76). A monocyte-derived ATMs expressing Lyve1 are recruited to hypoxic outgrowths of WAT in adolescent, lean mice. Lyve1+ ATMs express angiogenic matrix metalloproteinases (MMPs) and other angiogenic factors to form dense vascular networks that permit subsequent adipogenesis-mediated WAT development (63). MMPs degrade extracellular matrix components and are elevated in models of obesity. However, the extent of Lyve1+ ATM extracellular remodeling has yet to be explored. In addition, examples of macrophage-dependent angiogenesis in other contexts suggest M1 and M2 features are required for different stages of angiogenesis (77).

WAT requires extracellular remodeling to reduce stress on expanding and newly generated adipocytes and allow for proper vascularization. Unresolved remodeling and inflammation can lead to excessive extracellular matrix (ECM) component deposition, known as fibrosis. Obesity-induced WAT fibrosis has been linked to worsened metabolic dysfunction resistant to weight loss (78–80). In models of obesity-associated WAT fibrosis, macrophage depletion ameliorates fibrosis by reducing tissue inflammation and fibroblast activation (74). The primary mediator of macrophage-mediated activation of fibroblasts is TGF-β (63, 74, 81). In contrast, macrophages are also capable of ECM component uptake and degradation; however, these actions have not been explored in WAT.

Adipose tissue undergoes continual adipocyte turnover, refreshing the population through adipogenesis. In addition to adipogenesis within tissue outgrowths, ATMs initiate adipogenesis by expressing osteopontin to recruit pre-adipocytes towards a dying adipocyte (61, 64). In this way, the adipocyte is quickly replaced, and newly differentiated cells have space to develop; however, localization of the progenitor cell to the periphery of pro-inflammatory ATM clearance of a dead cell can be a double edge sword. Pro-inflammatory mediators such as TNF-α repress master adipogenic transcription factor PPARγ in pre-adipocytes (82, 83). During obesity, insufficient adipogenesis forces adipocytes to increase in size, or hypertrophy, and is correlated with increased inflammation, worsened metabolic health, and greater risk for co-morbidities (84–89).

## 4 Capturing dynamic ATM actions

Traditional methods to infer macrophage function rely on detecting a limited number of M1/M2 markers at the RNA or protein levels. Transcriptomic investigations, including microarrays and RNA-sequencing, have demonstrated the diversity of ATMs that cannot be neatly dichotomized into M1/M2 (15, 90) and revealed the importance of lipid-mediated reprogramming (15, 66). The advent of single-cell RNA-sequencing (scRNA-seq) has permitted unparalleled resolution to characterize the diversity in the immune compartment of adipose tissue.

### 4.1 Strategies in single-cell RNA-sequencing

ScRNA-seq generates highly dimensional data, and strategies to analyze begin with performing dimension reduction to facilitate downstream comparisons. Most strategies for dimension reduction utilize the whole transcriptome to establish similarities across cells, such as t-distributed stochastic neighbor embedding (tSNE) (91) and uniform manifold approximation and project (UMAP) (92). Following dimension reduction, cells are typically clustered into groups based on relative distances in the low-dimensional projection and overall transcriptomic similarity. These unsupervised algorithms delineate cells well based on major perturbations in the transcriptome to cluster cells based on lineage or for *de novo* subset identification. Researchers can then characterize clusters based on known cell markers and differential gene expression. However, due to frequent gene “dropouts” in scRNA-seq data, where expressed genes are not detected, and the variable turnover of marker proteins, traditional biomarker genes are not always reliable. To accommodate the disconnect observed between mRNA and protein levels, semi-supervised tools have been developed that use machine learning-based classifiers or manually curated lists of biomarkers to classify groups of cells based on their transcriptomic profiles (93–95). In addition, multiomic strategies combining scRNA-seq with targeted proteomic analysis (96) or spatial location (97) are becoming more popular. Application of scRNA-Seq in adipose tissue depicted the heterogeneity of ATMs, revealing the lipid-handling Tim4+ and obesity-associated Trem2+ population in mice (34, 62) and tissue immune cell dynamics across lineages in lean and obese humans (98).

Beyond identifying cell subsets, cell function is of great scientific interest in scRNA-seq studies. Several programs have been widely applied for the downstream cluster-based annotation of gene ontology from transcriptomics data, including DAVID (99, 100) and Qiagen Ingenuity Pathway Analysis (IPA) (101). However, these methods are not high-throughput and thus cannot represent the continuum of cell actions. In addition, while traditional whole-transcriptome dimension reduction and clustering techniques work well to distinguish cell lineages, differences in cell function, especially for cells such as macrophages, are often represented by much more subtle transcriptomic changes. Therefore, cell functional annotation in scRNA-seq data is a current technical challenge representing an area of active research.

### 4.2 Algorithms for function-guided cell annotation

Depicting the dynamic actions of macrophages in transcriptomics data has been challenging. Due to the whole-transcriptome input into unsupervised algorithms, functional distinctions are not intentionally utilized for clustering. Further, cells respond to diverse stimuli that induce divergent transcriptomes for multifaceted functions (102). Thus, capturing complex stimulation-induced signaling network changes towards multifaceted actions poses an important technical challenge in bioinformatics (103). To this end, we have designed two programs, MacSpectrum (90) and AtheroSpectrum (104), to depict three actions of macrophages along a spectrum of action intensity: monocyte maturation, macrophage inflammatory polarization, and atherosclerosis-related foaming. Applications of MacSpectrum in ATMs depicted the enrichment of pro-inflammatory ATMs in obesity (90), permitting investigation into obesity-associated macrophage inflammatory programs. Further, MacSpectrum characterized an ATM CD206+CD11c- subset enriched in diabetic obese humans as phenotypically distinct, with terminal differentiation and less pro-inflammatory than lipid-laden cells (105). AtheroSpectrum is tailored to atherosclerotic macrophages. Utilization of AtheroSpectrum revealed two novel macrophage foaming programs: homeostasis foaming and pathogenic foaming, the latter associated with cardiovascular disease. Depicting these distinct programs allowed for a focused investigation into pathogenic foaming that enabled leveraging program-specific genes to improve cardiovascular risk prediction models (104). These programs demonstrate the importance of depicting macrophage plasticity to parse out nuanced regulatory networks driving diversified macrophage function.

## 5 Discussion

Obesity is a major health risk in part due to adipose tissue dysfunction. In the adipose tissue, macrophages potentiate local inflammation through pro-inflammatory cytokine production and immune activation. However, ATMs represent a heterogeneous population that support tissue function through dead adipocyte clearance, lipid-buffering, extracellular remodeling, and supporting angiogenesis and adipogenesis. High-resolution techniques have allowed the identification of ATM subsets best suited to these functions. Leveraging scRNA-seq techniques to capture the spectrum of multifaceted macrophage actions allows for important macrophage programs to emerge that are not evident using traditional macrophage categorization models or low-resolution techniques. Function-guided macrophage annotations are important to understanding tissue heterogeneity and investigating ATM programs correlated with metabolic health.

## Author contributions

AM wrote and revised the manuscript. LQ, KK, AV and BZ contributed to manuscript design and revisions. All authors contributed to the article and approved the submitted version.
